# Natural Polymorphisms Are Present in the Furin Cleavage Site of the SARS-CoV-2 Spike Glycoprotein

**DOI:** 10.3389/fgene.2020.00783

**Published:** 2020-07-17

**Authors:** Yue Xing, Xiao Li, Xiang Gao, Qunfeng Dong

**Affiliations:** ^1^Department of Veterinary Integrative Biosciences, Texas A&M University, College Station, TX, United States; ^2^Department of Molecular and Cellular Medicine, Texas A&M University, College Station, TX, United States; ^3^Department of Medicine, Stritch School of Medicine, Loyola University Chicago, Maywood, IL, United States; ^4^Center for Biomedical Informatics, Stritch School of Medicine, Loyola University Chicago, Maywood, IL, United States

**Keywords:** COVID-19, SARS-CoV-2, spike glycoprotein, S protein, furin, mutation, live-attenuated vaccine

## Abstract

The furin cleavage site in the spike glycoprotein of the SARS-CoV-2 coronavirus is considered important for the virus to enter the host cells. By analyzing 45828 SARS-CoV-2 genome sequences, we identified 103 strains of SARS-CoV-2 with various DNA mutations including 18 unique non-synonymous point mutations, one deletion, and six gains of premature stop codon that may affect the furin cleavage site. Our results revealed that the furin cleavage site might not be required for SARS-CoV-2 to enter human cells *in vivo*. The identified mutants may represent a new subgroup of SARS-CoV-2 coronavirus with reduced tropism and transmissibility as potential live-attenuated vaccine candidates.

## Introduction

A notable feature of the SARS-CoV-2 coronavirus is that its spike glycoprotein contains a polybasic furin cleavage site at the S1-S2 boundary (Andersen et al., 2020; Walls et al., 2020). Furin is a protease ubiquitously expressed in multiple organs and tissues in humans, such as the brain, lung, gastrointestinal tract, liver, pancreas, and reproductive tissues (Wang et al., 2020). Cleavage of the spike protein by the furin protease is considered to facilitate the entrance of SARS-CoV-2 into host cells. Due to the wide expression of furin in multiple tissues, the existence of the furin cleavage site in the spike glycoprotein may expand tropism and enhance the transmissibility of SARS-CoV-2 (Walls et al., 2020).

When the discovery of the furin cleavage site was published on April 16, 2020 (Walls et al., 2020), there only existed 144 SARS-CoV-2 genome sequences in the GISAID database (Elbe and Buckland-Merrett, 2017; Shu and McCauley, 2017) and the furin cleavage site was strictly conserved (Walls et al., 2020). As of June 13, 2020, the number of SARS-CoV-2 genome sequences in the GISAID database has significantly increased to 45828. Therefore, we sought to answer a straightforward yet important question: are there any natural polymorphisms in the furin cleavage site of the SARS-CoV-2 spike glycoprotein? The existence of natural polymorphisms in the furin cleavage site may represent a new subgroup of SARS-CoV-2 coronavirus with different tropism and transmissibility.

## Methods

In total, 45828 SARS-CoV-2 genome sequences were downloaded from the GISAID database on June 13, 2020. The microbial genomics mutation tracker (MicroGMT) software, recently published by our group (Xing et al., 2020), was applied with default parameters to identify DNA mutations between each downloaded database sequence and the reference genome sequence of SARS-CoV-2 (i.e., SARA-CoV-2 isolate Wuhan-Hu-1 complete genome sequence with GenBank accession number NC_045512) (Wu et al., 2020). In brief, MicoGMT invokes (1) minimap2 (Li, 2018) to perform genome-wide pairwise alignments and (2) snpEff (Cingolani et al., 2012) to identify point mutations (synonymous and non-synonymous), insertions or deletions, and gains of stop codons from the genome alignments. The computation was performed for about 40 h in the high-performance research computer Ada at Texas A&M University. The NCBI Structure program^1^ was used to characterize the changes of the biochemical properties of non-synonymous mutations.

## Results

**FIGURE 1:**
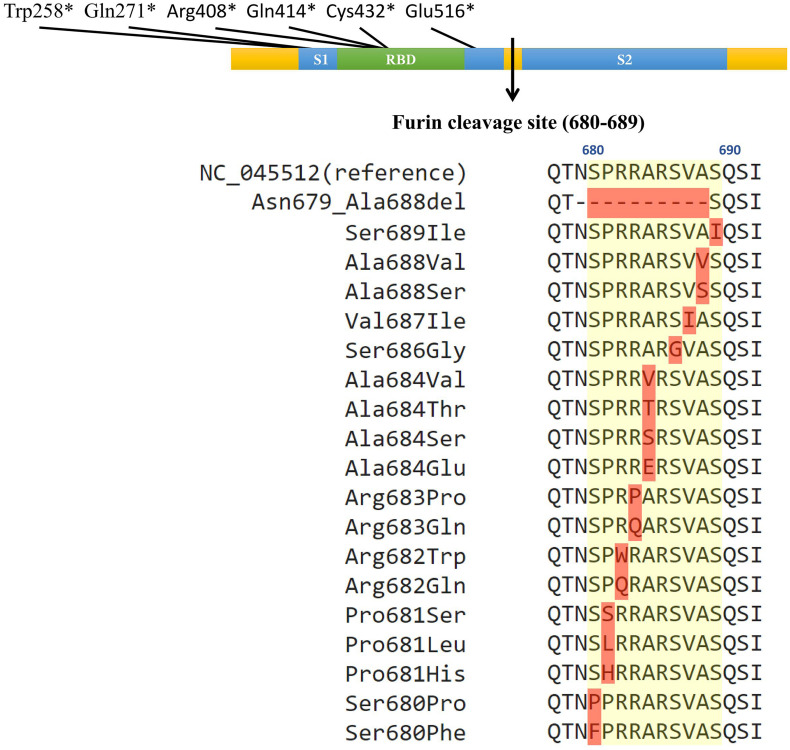
Naturally occurring polymorphisms in the furin cleavage site of the SARS-CoV-2 spike glycoprotein. The colored bar represents the spike glycoprotein, with the blue boxes indicating the S1 and S2 subunits, green box indicating the receptor binding domain, and the arrow indicating the furin cleavage site. The positions of the six gains of premature stop codons were illustrated on the top of the color bar (^∗^ indicates the stop codon). The yellow shade on the peptide sequences indicate the furin cleavage site, and red shades indicate mutations. The multiple sequence alignment shows each identified non-synonymous mutation with the yellow background indicating the furin cleavage site from the position 680 to 689.

From 45828 SARS-CoV-2 genome sequences available in the GISAID database as of June 13, 2020, 103 strains of SARS-CoV-2 carried various DNA mutations including 25 unique ones that may affect the furin cleavage site located at the amino acid residual positions 680–689 (S1/S2 region) (Coutard et al., 2020; Wang et al., 2020; Zhang et al., 2020) of the SARS-CoV-2 spike protein (Figure 1, Table 1, and Supplementary Table 1). Specifically, 96 SARS-CoV-2 strains were identified to carry a total of 23 unique point mutations in the furin cleavage site (each mutant strain carried only one non-synonymous point mutation in the furin cleavage site). Out of those 96 strains, 74 carried non-synonymous point mutations; out of the 23 unique point mutations, 18 were non-synonymous. Of those 18 non-synonymous changes, one changed from a non-polar amino acid residue (Ala) to a negatively charged residue (Glu); four changed from non-polar to neutral polar (Pro to Ser, Ala to Thr, and Ala to Ser at two different sites); one changed from non-polar (Pro) to positively charged (His); four changed from neutral polar to non-polar (Ser to Phe, Pro, Gly or Ile); two changed from positively charged to non-polar (Arg to Trp or Pro); two changed from positively charged to neutral polar (Arg to Gln at two different sites). Out of all the amino acid residues in the furin cleavage site, only Arg685 had no point mutations (neither synonymous nor non-synonymous). Besides point mutations, one strain (HongKong/XM-PII-S4/2020) contained a deletion in the furin cleavage site. The deletion spanned from Asn679 to Ala688, which is almost the entire length of the furin cleavage site (except for the last amino acid residue). In addition, we found six SARS-CoV-2 strains with the gains of stop codons in the spike protein between the position 258 and 516, which would abolish the downstream furin cleavage site located at the positions 680-689.

**TABLE 1:** Summary of mutations identified in the furin cleavage site.

Mutation	Type	Region	Biochemical property	Number of strains
Ser680Phe	Non-synonymous	North America	Polar but neutral to non-polar	1
Ser680Pro	Non-synonymous	North America	Polar but neutral to non-polar	1
Pro681His	Non-synonymous	Europe, North America	Non-polar to positively charged	3
Pro681Leu	Non-synonymous	Europe, North America	Both non-polar	23
Pro681Pro	Synonymous	Europe	N/A	4
Pro681Ser	Non-synonymous	Europe, Oceania	Non-polar to polar but neutral	5
Arg682Arg	Synonymous	Africa, Asia, Europe, North America	N/A	9
Arg682Gln	Non-synonymous	Asia, North America	Positively charged to polar but neutral	3
Arg682Trp	Non-synonymous	Asia, Europe, North America	Positively charged to non-polar	3
Arg683Arg	Synonymous	Asia, Europe	N/A	7
Arg683Gln	Non-synonymous	Europe	Positively charged to polar but neutral	2
Arg683Pro	Non-synonymous	Europe	Positively charged to non-polar	1
Ala684Ala	Synonymous	Asia	N/A	1
Ala684Glu	Non-synonymous	Asia	Non-polar to negatively charged	1
Ala684Ser	Non-synonymous	North America	Non-polar to polar but neutral	1
Ala684Thr	Non-synonymous	Europe, North America	Non-polar to polar but neutral	2
Ala684Val	Non-synonymous	Europe, South America	Both non-polar	7
Ser686Gly	Non-synonymous	Europe	Polar but neutral to non-polar	1
Ser686Ser	Synonymous	North America	N/A	1
Val687Ile	Non-synonymous	Europe	Both non-polar	1
Ala688Ser	Non-synonymous	Europe	Non-polar to polar but neutral	3
Ala688Val	Non-synonymous	Europe	Both non-polar	11
Ser689Ile	Non-synonymous	Europe	Polar but neutral to non-polar	5
Asn679_Ala688del	Deletion	Asia	N/A	1
Trp258*	Gain of stop codon	Asia	N/A	1
Gln271*	Gain of stop codon	Oceania	N/A	1
Arg408*	Gain of stop codon	Europe	N/A	1
Gln414*	Gain of stop codon	Oceania	N/A	1
Cys432*	Gain of stop codon	Asia	N/A	1
Glu516*	Gain of stop codon	Asia	N/A	1

* means stop codon.

The identified mutations in the furin cleavage site appeared in multiple geographic regions (Asia, Europe, North America, and Oceania) through January to May 2020. Most of them appeared in one or two geographic regions, but Arg682Trp appeared in three regions. Europe and North America had the most point mutations in the furin cleavage site. The only deletion was observed in Asia, and the gain of stop codons were observed in Asia, Europe, and Oceania.

## Discussion

We uncovered 103 SARS-CoV-2 strains from multiple geographic regions, 81 of which carried 25 unique mutations that may affect the furin cleavage site in the spike glycoprotein. Out of the total 10 amino acid residues in the furin cleavage site, nine experienced non-synonymous changes. It is worth noting that the non-synonymous point mutations occurred at seven out of eight amino acid residues of the highly conserved region of 682 RRARSVAS689 (Anand et al., 2020). This conserved region included three of the four amino acid residues of 681PRRA684 that are unique to SARS-CoV-2 (Zhang et al., 2020) (non-synonymous point mutations also occurred at Pro681), containing the furin cleavage point between Arg685 and Ser686 (Coutard et al., 2020). Although no mutations were identified at Arg685, mutations existed at Ser686 (e.g., Gly) disabling furin-type cleavages. In addition, mutations around Arg685 and Ser686 may also affect the recognition of the cleavage site. Point mutations and deletions were also found upstream and downstream of positions 680-689 including two deletions from position 675–679 (data not shown). Finally, we also observed one deletion and six gains of premature stop codons, all of which completely abrogated the furin cleavage site. Interestingly, Davidson et al. (2020) also detected one deletion in the furin cleavage site based on RNA-Seq sequencing.

Since all the mutations were identified from live viral strains in COVID-19 patients, our results revealed that the furin cleavage site may not be required for SARS-CoV-2 to enter human cells *in vivo*, which agrees with the *in vitro* experimental results showing that SARS-CoV-2, with deletion of the furin cleavage site, could still enter the cell lines of humans, African green monkeys, and bay hamsters (Walls et al., 2020). Therefore, we speculate that our observed mutants may represent a new subgroup of SARS-CoV-2 coronavirus with reduced tropism and transmissibility, which requires further experimental validations. Analyzing clinical symptoms and infectiousness of the COVID-19 patients with those mutant strains may be also important in future studies. If tropism and transmissibility of those mutant strains were indeed reduced, they might serve as potential live-attenuated vaccine candidates (Turell et al., 2003; Lauring et al., 2010; Toth et al., 2011).

## Data Availability Statement

All datasets presented in this study are included in the article/Supplementary Material.

## Author Contributions

XL and YX performed the data analysis and drafted the manuscript. XG and QD designed the project and revised the manuscript. All authors approved the submitted version.

## Conflict of Interest

The authors declare that the research was conducted in the absence of any commercial or financial relationships that could be construed as a potential conflict of interest.

## Footnotes

1 https://www.ncbi.nlm.nih.gov/Class/Structure/aa/aa_explorer.cgi
